# Bleaching Teeth

**Published:** 1878-09

**Authors:** 


### Editorial.

### "BLEACHING TEETH."

"*What is the best method of bleaching teeth?*"

This question is frequently repeated, not only by those afflicted, but by those who would remedy the difficulty.

The above query is just at hand from a dentist of acknowledged ability, and long experience. It is proposed in this instance, as doubtless it is in many others, not because the questioner is ignorant of any method of treating discolored teeth; he may be, and we presume is, familiar with good methods, but the question arises from a desire to know the best method, and that which will meet the greatest number of cases.

A full answer to this question involves many points, more, perhaps, than can be discussed here. This discoloration is not that which is found upon the surfaces of the teeth in the form of deposit. Rarely indeed does the enamel become stained or discolored beyond the surface. The exceptions are found only in enamel of defective structure, or when it has been deteriorated by some disintegrating agents.

The dentine is that part of the tooth usually involved in objectionable discoloration. This structure is variable in its susceptibility to coloring agents; in some cases readily taking up coloring material, in others resisting its entrance. This variation in susceptibility is dependent upon the condition of the dentine in respect to density or the opposite condition. In early life, or at any time before maturity, the teeth are more liable to discoloration than later in life. The age of the patient, therefore, is an important circumstance in the condition of the subject.

The teeth of different persons at the same time of life differ greatly, some being relatively quite soft and much more easily penetrated by foreign material than others; with others they are so dense and structurally so good that they become discolored with great difficulty, if at all. In almost all cases devitalization of the dentine takes place before it is discolored. There is an apparent, perhaps real, exception to this found in those instances in which decay has, by a change of condition, been arrested, the dentine in such a cavity of decay, is dark or even black, and still endowed with some sensitiveness in the dark part; this, however, is not often found.

In order to decide as to the proper course in any given case, the peculiarities and susceptibilities must be recognized and estimated.

A correct comprehension of the cause of the affection in question, is an important consideration, one that can not be overlooked if the best results are to be obtained.

The teeth are sometimes discolored by decomposition of the contents of the tubuli—the soft solid part of the structure. This is more liable to occur in the teeth of young persons, especially when the manifestation of the vital principle is feeble. This decomposition does not always produce a very marked discoloration, but it does frequently give a dark, muddy, unsightly color, that makes quite a contrast with a living, healthy tooth. This condition is liable to occur in any tooth that has lost its vitality whether decayed or not.

Again, discoloration of a red, purple or brown hue sometimes occurs by the entrance of the coloring matter of the blood, this can only take place where the constituents of the red corpuscles are separated and the cruorine in solution. This is the more likely to take place under stress of some great violence. The teeth of persons who have died from strangulation, by either hanging or drowning, are sometimes of a purple hue. Arsenious acid applied in small cavities for the removal of sensitiveness of dentine, will sometimes produce this discoloration. Instances of this mode of discoloration are far less frequent now than when arsenic was in common use for the treatment of sensitive dentine. The penetration of this material, like others, varies in different cases, sometimes being little more than superficial, at others, extending throughout the dentine, especially of the crown of the affected tooth.

The teeth often become discolored by the imbibition of foreign material that accumulates and is retained in cavities of decay till decomposition takes place.

The teeth are frequently discolored to a very marked degree by the compounds of oxydizable metals that are used for filling teeth. Rarely, if ever, is any compound of tin or silver taken up by dentine; they are never found in the mouth in a condition in which that can be done.

Mercury is largely used in compounds and mixtures for filling teeth, this, when in excess and subjected to the temperature of animal or blood heat, will pass into vapor. Mercury is volatile even at low temperatures, and quite so at the temperature of animal heat; its vapor is exceedingly subtle and penetrating. Mr. Henry Watts, in his Dictionary of Chemistry, vol. iii, p. 884, says: "Vapor rises from mercury between $+15.5°$ and $+27°$ (but not at $6.7°$) both in vacuum and in spaces filled with air, as shown by silvering of gold leaf kept for two months in a vessel over mercury." (Faraday, $+7°$.)

According to Karstan, mercury at a temperature below 0.° gives off sufficient vapor to bring out the image on a daguerreotype plate held over it.

Brame found that sulphur in the very finely divided utricular condition in which it is first precipitated from the state of vapor, is a much more delicate test for the presence of mercurial vapor than gold leaf. By means of this test he finds that at $+12°$ the vapor of mercury rises to a highth of more than a meter—that even at $+8°$ it appears to have no limited atmosphere; that it rises at ordinary temperatures from amalgams and mercurial ointment—that in presence of air and sulphur vapor it diffuses according to the same law as other gases."

Mercury as used in connection with other metals to form material for filling teeth, is in excess of the other metals employed; and in the mouth the conditions are favorable for the formation of vapor. Free mercury being present, and in a temperature of 38° it will necessarily vaporize; this vapor possesses great diffusibility, and will permeate almost any thing permeable, with which it may come in contact. The penetrating power of this vapor is greater than that of any other extraneous material with which the teeth are usually discolored. Its oxidation is readily affected when in contact with air or moisture, at ordinary temperatures; and the color then presented is that so often found in teeth that have been filled with amalgam. The discoloration from this material is sometimes found only upon the surface, but frequently it penetrates more or less deeply into the dentine, in some cases extending wholly through it, thus depending upon the susceptibility of the structure together with the prevention of the vapor from surface escape.

These are some of the ways in which teeth become discolored so as to render bleaching desirable.

How this may be best accomplished is the question.

There is no *single* method that is of universal application, that will accomplish the object in all cases. Cases of like forms and conditions, will be amenable to about an uniform treatment.

The first things, then, that claim attention upon the presentation of a case, are the character, condition and the cause of the discoloration. In the treatment of this discoloration, two modes of proceedure are available, viz: first, by the use of the proper cutting instruments remove the affected part; and second, by decomposition of the coloring material, which usually destroys its objectionable character.

By the first method, the removal of the dentine, as well as the coloring matter in it, is effected. This course is only practicable where the discoloration extends but a little way into the dentine, or when the body of dentine is sufficient to admit of the requisite cutting, for the removal of the objectionable part.

In all cases when the teeth would be much weakened by this mode, it should not be employed. But it is practicable when the offensive part may be cut away wholly, or as in ome cases, in part only, then by the introduction of some light substance that will reflect the light, neutralize the objectionable color in the tooth, to some extent at least.

Cutting away a part of the darkened dentine may so expose other parts that bleaching agents may be brought in contact with the substance to be acted upon.

When the employment of the second method, viz: decomposition, is decided upon, the following things should be noted: First, The nature of the substance to be decomposed. Second, The depth of its penetration. Third, The decomposing agent. Fourth, The method and facility of bringing the bleaching agent in contact with that upon which it is to act, viz: the coloring material. Fifth, The influence of the agent employed may have upon the tooth structure with which it may come in contact.

The different material with which the teeth are liable to be discolored, vary much in the facility with which they may be decomposed; some are permanent compounds, others feeble; some may be simply washed out, while others will resist the influence of every agent that is practicable in application; some are so stable that they remain unchanged though the tooth-bone in which they are lodged should be dissolved. Therefore a correct knowledge of the character of the material with which the teeth may be, or are, colored, is important to him who seeks its removal.

Teeth deeply stained by the coloring matter of the blood, are not easily freed from the discoloration, from, the fact that this stain seems to take a firm hold upon the tissue and agents that will decompose it will act upon the dentine as well.

The oxide of mercury, or indeed of any of the metals oxygenisable in the mouth, can not be decomposed in the substance of the teeth, nor indeed in contact with them, except by agents and processes that would at once be destructive to them. But to this statement the criticism may be made that cyanogen readily decomposes oxide of silver, and terchloride of gold, but to this it is only necessary to reply that the teeth are never stained with the gold-chloride, nor with oxide of silver, except upon the surface, and then cyanogen may be employed for its removal.

When the oxide of mercury, or any analogous substance has found a lodgment in the dentine, its removal is only practicable by cutting away the affected part. Chlorine and sulphurous acid are the chief bleaching agents. Cyanogen, while it acts with energy and promptness upon some substances, fails wholly to operate on others, and the danger in its use would forbid its general adoption.

Chlorine is perhaps more used in bleaching teeth, and rightly so, than any, or possibly all other agents put together. In speaking of this subject, Dr. Watt, in Chemical Essays, page 117, says: "Chlorine, which has been termed "the great bleaching agent," as is well known, has a strong affinity for hydrogen, and sometimes bleaches by taking this element from the coloring compound, and sometimes by taking hydrogen from the water present, thus liberating the oxygen, which in its nascent state is able to decompose the coloring matter by taking its hydrogen and corbon. In many instances of chlorine bleaching, the processes take place simultaneously, and are not in the least incompatible with each other.

"Oxygen, as it exists in the atmosphere, sometimes bleaches (and sometimes dyes) by virtue of its affinity for elements or compounds contained in the coloring matter."

In the employment of chlorine various processes have been adopted. Free chlorine has, by a properly formed and adjusted tube, been thrown into the decayed cavity of a tooth to be bleached, by this method the desired results have hardly been realized. Chlorinated fluids have been used, but generally with indifferent results.

The more frequent, and by far the best application, is the hypochlorite of lime. In Chemical Essays, page 127, Dr. Watt says: "In bleaching with this salt a number of reactions occur. The lime may be regarded as the pilot or engineer that conducts the acid to the place where the action is desired. Its direct action in promoting or retarding the bleaching process is of no practical importance. Chlorine has long been recognized as the great bleacher; but disputes have arisen as to how it bleaches, some maintain that by its affinity for hydrogen, it decomposes water, and the liberated oxygen, with the advantage of its nascent condition, does the bleaching. Others claim that it takes the hydrogen of the coloring principle and thus bleaches directly. But there is no occasion for dispute, for both positions are correct.

"It has been said the hypochlorite is constantly giving off the acid, and also that the acid itself is decomposed under all ordinary circumstances. Now bearing in mind that this acid is composed of one equivilent of oxygen and one of chlorine, by its decomposition their active elements are simultaneously liberated, having equally the advantage of the nascent state, and therefore are far more energetic than if previously free; the oxygen spends its force by taking the hydrogen and carbon from the coloring principle, while the chlorine either takes hydrogen from the coloring matter, or from the water present, in which case another equivalent of nascent oxygen is set to work.

"From this it is seen that one equivalent of hypochlorite of lime (containing, of course, one equivalent of hypochlorous acid) has as much bleaching power as two equivalents of free chlorine, or two of nascent oxygen."

There is therefore (perhaps) no better compound of chlorine than that with lime just referred to. Care, however, is requisite, lest by its too frequent application, serious injury is done to the tooth, one or two applications at most should be sufficient in any case. Another advantage possessed by chlorine over any other bleaching agent is its greater power of penetration, and so greater ability to reach the material upon which it is to act.

We trust that a careful consideration of the suggestions here made will lead in some measure at least to an understanding of the principles involved in treating discolored teeth.

